# Premature Atherosclerotic Cardiovascular Disease in a Middle Eastern Cohort: A Risk-Accumulation Perspective on Shared Determinants and Temporal Exposure Patterns

**DOI:** 10.3390/diagnostics16203354

**Published:** 2026-10-15

**Authors:** Osama Alkouri, Walid Al-Qerem, Salam Bani Hani, Ghaleb Alharbi, Eidan Alrasheid, Mohammad H. Abuadas, Abdulkareem Alshehri, Ahmad Saifan, Ayman Hammoudeh, Ahmad Y. Abuhelwa, Mohannad E. AbuRuz, Mohannad Alkhateeb, Nizar Alsubahi

**Affiliations:** 1 Faculty of Nursing, Yarmouk University, P.O. Box 566, Irbid 21163, Jordan; 2 Faculty of Pharmacy, Al-Zaytoonah University of Jordan, Amman 11733, Jordan; 3 Nursing Department, Nursing College, Al-Balqa Applied University, As Salt 19117, Jordan; 4 Department of Clinical Pharmacy, College of Pharmacy, Shaqra University, Shaqra 11961, Saudi Arabia; 5 Nursing Department, Ministry of Health, Kuwait City 13001, Kuwait; 6 Advanced Diagnostic and Therapeutic Institute, King Abdulaziz City for Science and Technology (KACST), P.O. Box 6086, Riyadh 11442, Saudi Arabia; 7 Department of Cardiology, Istishari Hospital, Amman 11184, Jordan; 8 Department of Pharmacy Practice and Pharmacotherapeutics, University of Sharjah, Sharjah 27272, United Arab Emirates; 9 Department of Health Service and Hospital Administration, Faculty of Economics and Administration, King Abdulaziz University, Jeddah 21589, Saudi Arabia; 10 School of Public Health and Community Medicine, Institute of Medicine, University of Gothenburg, 405 30 Gothenburg, Sweden; 11 Department of Health Services Research, Care and Public Health Research Institute (CAPHRI), Faculty of Health, Medicine and Life Sciences, Maastricht University Medical Center, Maastricht University, 6200 MD Maastricht, The Netherlands

**Keywords:** premature atherosclerotic cardiovascular disease, risk factor accumulation, atherogenic dyslipidemia, cardiovascular risk stratification, Middle Eastern population

## Abstract

**Background:** Premature atherosclerotic cardiovascular disease (ASCVD) represents a growing clinical and public health concern, particularly in regions with a high burden of modifiable risk factors. Whether premature ASCVD reflects a distinct pathophysiological entity or an earlier manifestation of shared risk exposures remains incompletely understood. This study aimed to characterize the clinical profile of premature ASCVD and to examine its relationship with conventional cardiovascular risk factors within a Middle Eastern cohort. **Methods:** This pooled observational analysis included 2763 adults with established ASCVD derived from multiple cardiovascular registries and a prospective cohort. Premature ASCVD was defined using sex-specific age thresholds (<55 years in men and <65 years in women). Multivariable logistic regression analyses, including sex-stratified models, were performed to evaluate associations between premature presentation and cardiovascular risk factors, including smoking, family history, lipid parameters, and cardiometabolic comorbidities. **Results:** Among 2763 participants in the pooled clinical cohort, 1335 (48.3%) met the study definition of premature ASCVD. Compared with later-onset disease, premature ASCVD was strongly associated with current smoking, family history of premature cardiovascular disease, and a more atherogenic lipid profile, including higher levels of low-density lipoprotein cholesterol, triglycerides, and non-high-density lipoprotein cholesterol. In contrast, later-onset ASCVD was characterized by a higher prevalence of hypertension and diabetes mellitus. These patterns were consistent across sex-stratified analyses. Despite high rates of secondary prevention therapy use, a substantial residual atherogenic burden was observed among patients with premature ASCVD. **Conclusions:** In this predominantly coronary cohort, the findings suggest that premature and later-onset ASCVD occur within a broadly overlapping risk-factor landscape, but that the relative distribution of these factors differs according to age at first clinical presentation. The greater prevalence of smoking, family history, and atherogenic lipid abnormalities among younger patients may reflect earlier or greater exposure to these factors, whereas the greater burden of hypertension, diabetes mellitus, and other chronic comorbidities among older patients is consistent with their progressive accumulation with age. These cross-sectional associations should not be interpreted as demonstrating distinct causal mechanisms or age-independent risk profiles.

## 1. Introduction

Atherosclerotic cardiovascular disease (ASCVD) remains a leading cause of morbidity and mortality worldwide, but its burden is not distributed equally across populations or regions [1,2]. The Middle East and North Africa region has experienced a substantial cardiovascular disease burden, shaped by demographic transition, high prevalence of conventional risk factors, changing dietary and lifestyle patterns, and persistent gaps in early prevention [3]. Within this broader regional burden, ASCVD occurring at younger ages is particularly important because it affects individuals during economically and socially productive years and may confer a longer lifetime exposure to recurrent cardiovascular events, disability, and long-term pharmacotherapy.

Premature ASCVD is commonly defined using sex-specific age thresholds, typically before 55 years in men and before 65 years in women [4,5]. Clinically, premature ASCVD is important because it may occur before conventional short-term risk prediction tools identify an individual as being at high absolute risk, particularly when the main drivers are cumulative tobacco exposure, familial susceptibility, or severe lipid abnormalities rather than older age or established cardiometabolic comorbidity [5,6]. In this context, advanced noninvasive imaging modalities, including coronary computed tomography angiography (CCTA) and carotid ultrasound, have emerged as pivotal tools for shifting the preventive paradigm toward proactive, personalized risk stratification before clinical events [7].

The risk-factor architecture of premature ASCVD may differ from that of later-onset disease. In the INTERHEART study, a small group of modifiable risk factors, including abnormal lipids, smoking, hypertension, diabetes, abdominal obesity, diet, physical activity, alcohol intake, and psychosocial factors, accounted for most of the population-attributable risk of myocardial infarction across diverse world regions [8]. However, the relative expression of these risk factors may vary by age at presentation. Younger patients with ASCVD are often enriched for active smoking, family history of premature cardiovascular disease, and lipid abnormalities, whereas later-onset ASCVD is more frequently accompanied by hypertension, diabetes mellitus, chronic kidney disease, heart failure, and other comorbidities that accumulate with age [6,9]. Thus, premature ASCVD may represent an earlier clinical manifestation of the broader ASCVD spectrum in which the relative contribution and cumulative exposure to individual risk factors vary with age at presentation.

This question is especially relevant in Middle Eastern populations, where regional studies link premature myocardial infarction and acute coronary syndrome to a higher prevalence of smoking but a lower prevalence of diabetes and hypertension compared with older patients, alongside a distinct contribution of family history, dyslipidemia, and sex-specific risk patterns [9]. Nevertheless, much of this evidence has focused on acute coronary syndrome or coronary artery disease alone, leaving premature ASCVD across a broader spectrum of established atherosclerotic disease, with detailed lipid phenotyping, comparatively understudied.

Despite the clinical importance of premature ASCVD, data describing its clinical and lipid phenotype in Middle Eastern patients with established ASCVD remain limited. Better characterization of this phenotype may help identify patients and families who require earlier preventive intervention, more intensive lipid evaluation, and targeted tobacco-control strategies. Therefore, in this pooled Middle Eastern cohort of adults with established ASCVD, we aimed to determine the prevalence of premature ASCVD using sex-specific age thresholds, compare the clinical and lipid phenotype of premature versus later-onset ASCVD, and identify sex-specific factors associated with premature presentation.

## 2. Materials and Methods

### 2.1. Study Design and Data Sources

This study employed a pooled observational design, integrating data from harmonized clinical datasets of adults (≥18 years) with established atherosclerotic cardiovascular disease (ASCVD). The analytic dataset combined information from a prospective cohort and multiple established cardiovascular registries, all of which utilized comparable case-report forms and standardized clinical definitions. Data were merged following harmonization of variable coding and eligibility criteria to ensure consistency across sources.

Despite the differences in the primary aim of each study, time frame, and inclusion criteria, all made use of standard case report forms, similar definitions of risk factors and cardiovascular outcomes, and overlapping health facilities from Jordan and the larger region of the Middle East.

Because the contributing datasets differed in study objectives, recruitment periods, clinical settings, and eligibility criteria, the pooled cohort was considered a heterogeneous clinical sample rather than a population-representative ASCVD population. Pooling was restricted to variables that could be defined and measured consistently across datasets; harmonization improved coding comparability but did not eliminate differences in sampling frames.

Registry/source was not included as a conventional covariate because it was closely linked to study-specific selection mechanisms, including age- and sex-specific recruitment, long-term post-revascularization survival, and specific clinical presentations. Source adjustment therefore could not fully account for the underlying selection differences and could overlap with variables defining the source populations. The analysis was consequently intended to examine associations between selected risk factors and age at first documented ASCVD presentation within the assembled cohorts, rather than estimate population prevalence or causal effects. These limitations were considered when interpreting prevalence estimates and between-group comparisons.

The prospective component comprised participants enrolled in the Jordan Standard Modifiable Risk Factors (SMuRF)-less Study (ClinicalTrials.gov Identifier: NCT06199869) between January 10 and 20 August 2024, recruited from nine healthcare institutions across Jordan, including a private academic center, Ministry of Health–affiliated hospitals, community-based facilities, and university-affiliated hospitals. The retrospective component involved a post hoc analysis of established registries across the Middle East, including the Jordan Percutaneous Coronary Intervention Registry (NCT01841346) [10], the Surviving a Decade or More after Coronary Revascularization Study (NCT03491722) [10], the Jordan Atrial Fibrillation Study (NCT03917992) [11], the Jordan COVID-19 Pandemic Acute Cardiovascular Events Study (NCT04368637) [11], and the Study of Novel and Classical Risk Factors in Young Middle Eastern Women with ASCVD (NCT04975503) [11] (Table 1).

Data were collected using standardized case-report forms by trained research personnel to ensure methodological rigor and data quality. Variables included demographic and anthropometric characteristics, clinical history, conventional and emerging cardiovascular risk factors (modifiable and non-modifiable), comorbidities, use of secondary prevention therapies, and one-year post-event survival outcomes.

### 2.2. Study Population

The pooled source population included 2763 adults with ASCVD. Eligible participants were adults aged 18 years or older with clinically documented ASCVD. ASCVD was defined as documented coronary artery disease, ischemic stroke, peripheral artery disease, or carotid artery atherosclerosis. Coronary artery disease included chronic stable angina, acute coronary syndromes, and coronary artery disease diagnosed by coronary computed tomography angiography. Participants were classified as having premature ASCVD or later-onset ASCVD according to sex-specific age thresholds. Crucially, premature status was determined strictly based on the patient’s age at the time of their first documented clinical ASCVD event (the index event), rather than their age at study enrollment. This index-event age was ascertained identically across all five pooled data sources (Table 1), using documented diagnosis or procedure dates in the medical record rather than the date of registry entry. Premature ASCVD was defined as an index event occurring before 55 years of age in males and before 65 years of age in females, while later-onset ASCVD included all participants presenting at or beyond these thresholds. These thresholds follow current American College of Cardiology (ACC)/American Heart Association (AHA) and European Society of Cardiology (ESC)/European Atherosclerosis Society (EAS) guideline definitions of premature ASCVD [4,5] and were adopted to maintain consistency with prevailing clinical and risk-assessment frameworks, rather than an empirically derived or study-specific cutoff.

ASCVD ascertainment was based on diagnoses recorded during routine hospital care, where the diagnostic approach followed international clinical guidelines. Detailed coronary angiographic measurements and the investigation results supporting peripheral artery disease (PAD) and carotid atherosclerosis diagnoses were not available in the pooled analytical dataset. Consequently, the application of uniform condition-specific diagnostic thresholds could not be independently verified from these data.

### 2.3. Variables Collected and Definitions

Clinical and biochemical variables were defined using standardized criteria and analyzed in an order consistent with their presentation in descriptive and regression analyses. Demographic and behavioral variables included sex and current smoking status. For analysis, “smoking” was defined operationally as use of tobacco regularly during the last 12 months before enrollment into the study. The duration of 12 months was used as an operational time period for gathering smoking data from the source registries. This particular choice of operational definition was required due to the way in which the data were collected in the sources and not because of any common definition of “current smokers.” Since smoking was measured as one variable in the source registries, the distinction could not be made between cigarette smoking and waterpipe (shisha) [12]. Family history of premature cardiovascular disease was defined as cardiovascular disease occurring before 55 years of age in a first-degree male relative or before 65 years of age in a first-degree female relative [4,5,13,14,15]. Cardiometabolic clinical variables included hypertension, diabetes mellitus, dyslipidemia, body mass index (BMI), overweight, obesity, chronic kidney disease, and heart failure. Hypertension was defined by prior diagnosis, antihypertensive treatment, or persistently elevated blood pressure (≥140/90 mmHg) [16]. Diabetes mellitus was defined by medical history, use of glucose-lowering therapy, or HbA1c ≥ 6.5% [17]. Dyslipidemia was identified based on prior diagnosis, lipid-lowering therapy, or elevated LDL-C levels [18]. BMI was calculated as weight in kilograms divided by height in meters squared (kg/m^2^), with overweight and obesity categorized according to recorded BMI values [19]. Chronic kidney disease and heart failure were defined based on documented clinical diagnoses in the source datasets.

Lipid variables included low-density lipoprotein cholesterol (LDL-C), high-density lipoprotein cholesterol (HDL-C), triglycerides, total cholesterol, non–HDL-C, and the total cholesterol/HDL-C ratio. LDL-C was analyzed both continuously and categorically using clinically relevant thresholds of ≥190 mg/dL and ≥100 mg/dL. Triglycerides were analyzed continuously and as a binary variable using a threshold of ≥150 mg/dL, consistent with guideline definitions of hypertriglyceridemia [4]. Low HDL-C was defined as <40 mg/dL in men and <50 mg/dL in women, consistent with National Cholesterol Education Program Adult Treatment Panel III (NCEP ATP III) criteria [20]. Non–HDL-C was calculated as total cholesterol minus HDL-C and reflects total atherogenic lipoprotein burden [14,21]. The total cholesterol/HDL-C ratio was included as an index of the balance between atherogenic and protective lipoproteins [22].

ASCVD presentation and diagnostic categories included acute coronary syndrome (ACS), chronic stable angina, coronary artery disease diagnosed by CCTA, cerebrovascular accident, and peripheral artery disease (PAD). ACS was defined as acute myocardial ischemia, including ST-elevation and non–ST-elevation myocardial infarction. Metabolic syndrome was not modeled as a composite variable; instead, its individual components (hypertension, diabetes mellitus, obesity, triglycerides, and HDL-C) were analyzed separately to better characterize cardiometabolic burden. Secondary prevention medications, including antiplatelet agents, statins, beta-blockers, and renin–angiotensin–aldosterone system inhibitors, were recorded as present or absent at the time of data collection.

### 2.4. Ethical Considerations

This study was conducted in accordance with the ethical principles of the Declaration of Helsinki. It was approved by the Institutional Review Board/Independent Ethics Committee of Istishari Hospital, Amman, Jordan. The study protocol was registered on ClinicalTrials.gov (Identifier: NCT06199869). The data analyzed were obtained from a pooled database of 5540 patients enrolled in previously published cardiovascular studies, all registered on ClinicalTrials.gov and conducted by the same investigative group. For the prospective cohort (Jordan SMuRF-less Study), all participants provided written informed consent before enrollment. For the retrospective registries, informed consent was obtained at the time of enrollment in each original study, as documented in their respective approved protocols. For the current pooled analysis, the Institutional Review Board granted a waiver of additional patient consent because the analysis involved secondary use of fully de-identified data from previously completed studies under the custodianship of the same authors, posed minimal risk to participants, and re-contacting participants was deemed impracticable.

### 2.5. Data Harmonization

Because the study pooled data from multiple registries and a single prospective cohort, variables were harmonized before analysis. Harmonization included aligning variable names, unifying categorical coding, and applying the same clinical definitions across sources whenever possible. Variables that could not be consistently harmonized across datasets were excluded from the pooled analysis. Participants with missing essential variables, including lipid measurements or other key covariates needed for the final models, were excluded from the analytic cohort. To address between-source heterogeneity as a result of differences in time period for recruitment, context of care, and purpose of study, two steps were undertaken. First, all variables for which the definition and methodology of data collection were the same for each source were used, whereas variables that could not be harmonized were excluded from analyses rather than estimated. Second, in view of the fact that the analysis aimed to explore the cross-sectional relationship of risk factors to premature ASCVD, and not to provide an estimation of prevalence or treatment effect, registry source was not entered as a covariate.

To be completely consistent with the prospective cohorts and retrospective registries that contain long-term survivors (Survival Post-Coronary Revascularization), the age at first clinical manifestation contained in the baseline medical records of each data set was reviewed to determine the actual age at baseline. This age at event at baseline was considered the absolute standard for grouping and prevented survivor bias or delayed recruitment from classifying past cases prematurely as later-onset cases. In other words, consistent with the age-at-event definition described in Section 2.2, premature status was determined using each patient’s age at their first documented clinical ASCVD event (the index event)—not age at registry enrollment or data collection. For the retrospective registries, including the Survival Post-Coronary Revascularization cohort of long-term survivors, this index-event age was extracted from baseline medical records rather than inferred from enrollment date, preventing delayed recruitment from causing misclassification of patients with a premature index event as later-onset disease. However, accurate ascertainment of index-event age does not eliminate survivorship or selection bias inherent to the long-term survivor cohort.

### 2.6. Lipid Measurements and Statin Therapy

Most lipid measurements (*n* = 2636; 95.4%) were obtained before initiation of lipid-lowering therapy, while the remaining measurements (*n* = 127; 4.6%) were collected 2–4 weeks after starting statin treatment. Secondary prevention medication use was recorded “.at the time of data collection, which in most cases followed the index ASCVD event. Statin intensity was not reported because treatment decisions, including dose and intensity, were not standardized and were left to the discretion of the treating physicians, resulting in substantial variability that could not be reliably categorized for analysis. While the first definition is an inclusive one that includes patients with clinically diagnosed or treated dyslipidemias irrespective of the LDL-C level, the latter definition points to a narrower phenotype of hypercholesterolemia that meets the clinical guidelines for severity, which are unrelated to either a diagnosis or treatment of lipid disorders. The two measures differ by conceptual meaning, yet partially overlap as far as patient population is concerned.

### 2.7. Statistical Analysis

Categorical variables were summarized as *n*/N (%), and continuous variables as medians and interquartile ranges. Standardized mean differences were used to quantify between-group differences. Categorical variables were compared using Pearson’s chi-square test, and continuous variables using the Mann–Whitney U test. Sex-stratified comparisons were conducted separately within males and females. Each comparison used participants with available data for the variable concerned. Age was summarized descriptively without hypothesis testing because it determined premature versus later-onset classification. *p* values were two-sided and interpreted as exploratory, without adjustment for multiple comparisons.

Because the definition of premature ASCVD uses different age thresholds for males and females, the primary adjusted analysis was stratified by sex rather than relying on a pooled sex coefficient. Age was not entered as an adjusted covariate because it is embedded in the outcome definition. Sex-stratified logistic regression models estimated adjusted odds ratios (ORs) and 95% confidence intervals (CIs) for premature ASCVD. Covariates were selected a priori on clinical grounds and entered simultaneously into the models, without univariable significance screening or automated stepwise selection. The core models included family history of premature cardiovascular disease, current smoking, hypertension, dyslipidemia, diabetes mellitus, LDL-C ≥ 190 mg/dL, and ACS presentation. Supplementary clinical subset models additionally included overweight, obesity, chronic kidney disease, and heart failure; these models used participants with complete data for the included variables. Potential multicollinearity among covariates in the sex-stratified core logistic regression models was assessed using variance inflation factors (VIFs) and corresponding tolerance values. A VIF <5 and tolerance >0.20 were considered to indicate the absence of problematic multicollinearity. Statistical analyses were performed using Python version 3.12.14 and R version 4.5.3.

## 3. Results

### 3.1. Study Population and Baseline Characteristics

**Figure 1:**
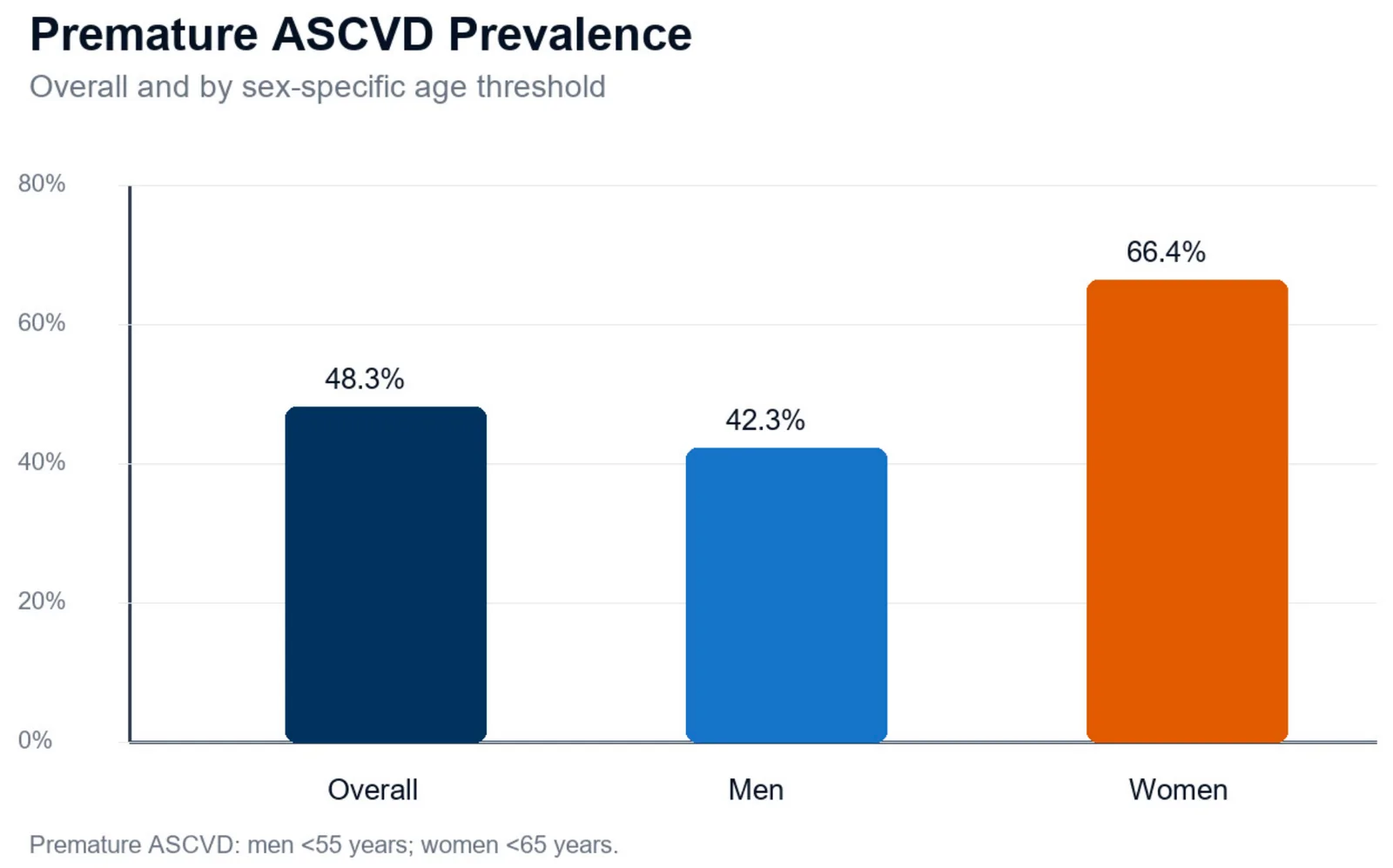
Proportion of Participants Classified as Having Premature ASCVD Overall and by Sex in the Pooled Clinical Cohort. Premature ASCVD was defined as an index event before age 55 years in males and before age 65 years in females. These proportions reflect the composition of the pooled study cohorts and are not population-prevalence estimates.

The analytic cohort included 2763 adults with established ASCVD, with a median age of 57.0 years (IQR, 49.0–65.0). The sample comprised 2073 males (75.0%) and 690 females (25.0%). Overall, 1335 participants (48.3%) had premature ASCVD and 1428 (51.7%) had later-onset ASCVD. Premature ASCVD was identified in 877/2073 males (42.3%) and 458/690 females (66.4%). These proportions describe the composition of the pooled study population and should not be interpreted as sex-specific population prevalence estimates, particularly because one contributing study specifically enrolled young Middle Eastern women, as summarized in Figure 1.

### 3.2. Comparison of Premature and Later-Onset ASCVD

Compared with patients with later-onset ASCVD, those with premature ASCVD were more often female (34.3% versus 16.2%) and had a substantially younger median age (49.0 years [IQR, 44.0–53.0] versus 65.0 years [IQR, 59.0–71.0]). Current smoking (57.2% versus 39.8%) and family history of premature cardiovascular disease (48.5% versus 37.4%) were more frequent in the premature ASCVD group. In contrast, hypertension (46.4% versus 67.0%) and diabetes mellitus (48.6% versus 58.5%) were more common in later-onset ASCVD. Additionally, the lipid profile also differed between groups. Premature ASCVD was associated with higher LDL-C, with a median LDL-C of 116.0 mg/dL [IQR, 89.0–148.5] compared with 100.0 mg/dL [IQR, 77.0–128.0] in later-onset ASCVD. LDL-C >= 190 mg/dL was observed in 7.6% of premature cases compared with 4.0% of later-onset cases, and LDL-C >= 100 mg/dL was present in 66.6% versus 51.5%, respectively. Premature ASCVD was also accompanied by higher triglycerides, higher non-HDL-C, and a higher HDL-C ratio. Median non-HDL-C was 145.0 mg/dL [IQR, 115.0–181.0] in premature ASCVD and 125.0 mg/dL [IQR, 99.0–156.0] in later-onset ASCVD, while the median total cholesterol/HDL-C ratio was 5.0 [IQR, 3.9–6.3] versus 4.4 [IQR, 3.5–5.6], respectively (Table 2 and Table 3).

Sex-stratified descriptive findings showed that smoking was the most prominent behavioral feature of premature ASCVD, particularly among males. Among males, current smoking was present in 74.7% of premature cases compared with 46.3% of later-onset cases. Among females, current smoking was less common overall but remained more frequent in premature ASCVD than in later-onset ASCVD (23.8% versus 6.5%). Family history of premature cardiovascular disease followed the same direction in both sexes, occurring in 49.7% of premature males versus 37.9% of later-onset males and in 46.1% of premature females versus 34.9% of later-onset females. Hypertension and diabetes mellitus were consistently more frequent in later-onset disease in both sexes (Table 4).

The recorded diagnostic categories comprised ACS in 2378 participants (86.1%), chronic stable angina in 261 (9.4%), coronary artery disease (CAD) diagnosed by CCTA in 66 (2.4%), cerebrovascular accident in 48 (1.7%), and PAD in 10 (0.4%). Their distributions by premature and later-onset ASCVD status are presented in Supplementary Table S1. Carotid atherosclerosis was not separately coded in the diagnostic classification and could not be quantified independently.

Secondary prevention medication use was high in both groups, with statin use recorded in 93.4% of premature ASCVD and 94.7% of later-onset ASCVD. Any antiplatelet therapy and the combined statin plus antiplatelet pattern were also frequent in both groups (Supplementary Table S2).

### 3.3. Multivariable Analyses

**Figure 2:**
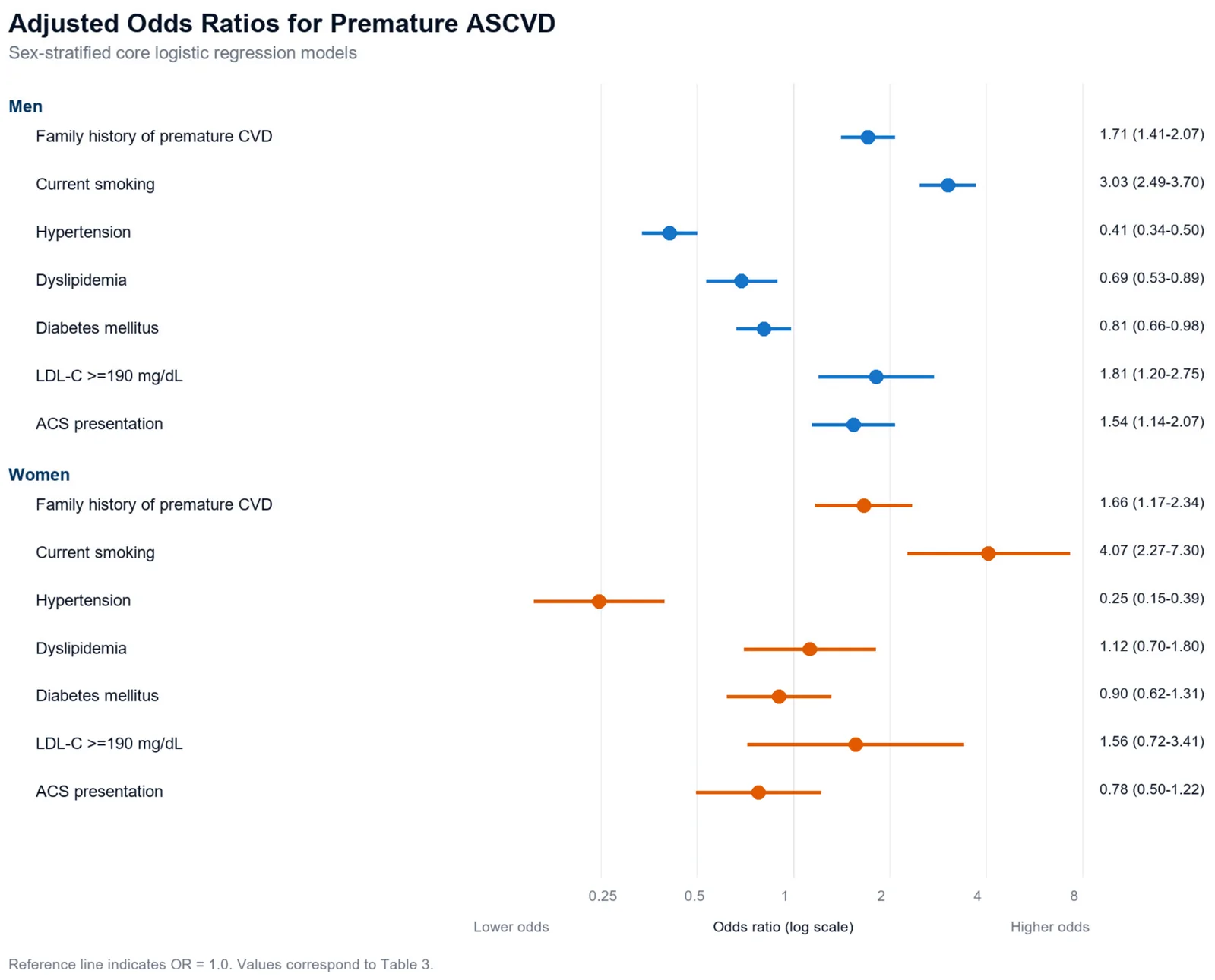
Adjusted Odds Ratios for Premature ASCVD in Sex-Stratified Core Models. Note. The reference line indicates OR = 1.0.

In sex-stratified adjusted models, current smoking and family history of premature cardiovascular disease were consistently associated with premature ASCVD, with the adjusted associations displayed in Figure 1. Among males, current smoking was associated with threefold higher odds of premature ASCVD (OR, 3.03; 95% CI, 2.49–3.70), while family history was associated with higher odds of premature presentation (OR, 1.71; 95% CI, 1.41–2.07). LDL-C >= 190 mg/dL was also associated with premature ASCVD in Males (OR, 1.81; 95% CI, 1.20–2.75), as was ACS presentation (OR, 1.54; 95% CI, 1.14–2.07). Hypertension, dyslipidemia, and diabetes mellitus showed inverse associations with premature presentation among males (Table 5 and Figure 2).

Among females, current smoking showed the strongest association with premature ASCVD (OR, 4.07; 95% CI, 2.27–7.30), and family history of premature cardiovascular disease was also associated with premature presentation (OR, 1.66; 95% CI, 1.17–2.34). Hypertension was inversely associated with premature ASCVD (OR, 0.25; 95% CI, 0.15–0.39. Dyslipidemia, diabetes mellitus, LDL-C >= 190 mg/dL, and ACS presentation were not independently associated with premature ASCVD among females in the core adjusted model (Table 3 and Figure 2).

Formal collinearity assessment showed no evidence of problematic multicollinearity in either sex-stratified core model. VIFs ranged from 1.03 to 1.30 among males and from 1.02 to 1.21 among females; corresponding tolerance values ranged from 0.77 to 0.97 and from 0.83 to 0.98, respectively.

The supplementary regression model (Supplementary Table S3) was consistent with the primary model while additionally accounting for obesity, chronic kidney disease, and heart failure. Among men, smoking and family history continued to show strong associations with premature ASCVD, and obesity was also linked to greater odds of premature presentation (OR, 1.86; 95% CI, 1.34–2.59). Chronic kidney disease, however, was associated with lower odds of premature ASCVD in men (OR, 0.44; 95% CI, 0.25–0.78). Among women, smoking and family history remained associated with premature presentation, whereas heart failure showed an inverse association with premature ASCVD (OR, 0.59; 95% CI, 0.36–0.96). As with hypertension and diabetes mellitus in the primary model.

## 4. Discussion

In this pooled observational analysis, premature ASCVD was common and reflected differences in the relative burden and timing of shared cardiovascular risk factors rather than a distinct disease phenotype. Premature presentation was more strongly characterized by smoking, family history, and atherogenic lipid burden, whereas later-onset disease showed greater hypertension and diabetes mellitus, with similar patterns for chronic kidney disease and heart failure. Because premature ASCVD was defined by age at first event (<55 years in men; <65 years in women), age was inherently embedded in the outcome and was therefore excluded from the primary regression models. The greater cardiometabolic comorbidity in later-onset disease likely reflects age-related risk accumulation rather than protective effects.

Overall, the findings support a temporal-exposure framework in which premature and later-onset ASCVD share common risk factors but differ in their relative burden and cumulative exposure. Given the observational, case-only design, these differences should not be interpreted as evidence of distinct disease mechanisms.

### 4.1. Prevalence and Sex Distribution of Premature ASCVD

Premature ASCVD was identified in 48.3% of the cohort (42.3% of males and 66.4% of females). This reflects a selected population with established ASCVD, predominantly acute coronary syndrome, rather than population prevalence in Jordan or the wider Middle East. Because non-coronary presentations were uncommon, the findings are most applicable to premature coronary/ACS disease.

This high proportion is consistent with the substantial ASCVD burden in the Middle East and North Africa, where smoking, dyslipidemia, obesity, diabetes, and changing lifestyles contribute to cardiovascular risk [9,23]. Regional evidence similarly links premature myocardial infarction and ACS to smoking and other modifiable factors, whereas diabetes and hypertension are more prevalent among older patients [9].

The higher proportion of premature ASCVD among females should be interpreted cautiously. It does not indicate earlier disease onset in women but partly reflects the sex-specific definition (<65 years in women vs. <55 years in men) [4,5], which encompasses a wider age range in women. Sex-stratified analyses therefore provide a more appropriate framework for interpreting these associations.

### 4.2. Smoking as the Dominant Modifiable Risk Factor Associated with Premature ASCVD

Current smoking was the strongest modifiable factor associated with premature ASCVD in both sexes, with approximately threefold higher odds in men (OR, 3.03; 95% CI, 2.49–3.70) and fourfold higher odds in women (OR, 4.07; 95% CI, 2.27–7.30). Smoking prevalence was also substantially higher among those with premature versus later-onset ASCVD in both men (74.7% vs. 46.3%) and women (23.8% vs. 6.5%).

These findings are consistent with evidence linking tobacco exposure to accelerated atherosclerosis and acute cardiovascular events through endothelial dysfunction, oxidative stress, inflammation, platelet activation, and impaired fibrinolysis [24,25]. INTERHEART similarly demonstrated a strong dose-response association between smoking and myocardial infarction, particularly among younger men [25]. Although the higher odds ratio in women should be interpreted cautiously given the smaller number of female smokers and absence of a formal sex-by-smoking interaction analysis, prior evidence suggests that smoking may confer greater relative coronary risk in women [26].

The findings are particularly relevant in Jordan, where the 2019 National STEPwise Survey reported smoking in 41% of adults (65.3% of men and 16.4% of women) [27]. These findings underscore the importance of smoking assessment and cessation interventions in preventing premature ASCVD in Jordan and similar Middle Eastern settings.

### 4.3. Family History and Inherited Susceptibility of Premature ASCVD

Family history of premature cardiovascular disease was consistently associated with premature ASCVD in both sexes, with higher adjusted odds in men (OR, 1.71; 95% CI, 1.41–2.07) and women (OR, 1.66; 95% CI, 1.17–2.34). Approximately one-half of patients with premature ASCVD reported a positive family history compared with 37–38% of those with later-onset disease (SMD, 0.22).

Family history may reflect inherited and shared environmental susceptibility, including familial hypercholesterolemia (FH), polygenic risk, elevated lipoprotein(a), and shared cardiometabolic or lifestyle factors. Accordingly, guidelines recognize premature ASCVD in the family as a risk-enhancing factor warranting earlier and more intensive risk assessment [4,5].

LDL-C ≥ 190 mg/dL was more frequent in premature than later-onset ASCVD (7.6% vs. 4.0%), supporting possible severe hypercholesterolemia or FH in a subset. This threshold warrants intensive lipid-lowering therapy and consideration of FH evaluation, particularly with premature ASCVD or positive family history [4,18,28]. However, because genetic testing and formal FH diagnostic criteria were not used, the study cannot establish FH prevalence or causality. These findings nevertheless support systematic lipid evaluation and family-based risk assessment in patients with premature ASCVD [28].

### 4.4. Atherogenic Dyslipidemia and the LDL-C Burden

The lipid profile showed a distinct atherogenic pattern in premature ASCVD. Median LDL-C was higher in premature than later-onset patients (116.0 vs. 100.0 mg/dL; SMD, 0.36), and LDL-C ≥ 190 mg/dL was more frequent (7.6% vs. 4.0%). This threshold indicates severe hypercholesterolemia and may suggest familial hypercholesterolemia, particularly with premature ASCVD or a positive family history, although it is not diagnostic alone [4,18,28]. Premature ASCVD was also associated with higher triglycerides, non-HDL-C, and TC/HDL-C ratio, whereas HDL-C differed minimally. These findings are consistent with evidence linking dyslipidemia to premature myocardial infarction and young-onset ASCVD and support assessment of overall atherogenic lipid burden rather than a single lipid threshold [14,18,22,29].

LDL-C ≥ 190 mg/dL was associated with premature ASCVD in men (OR, 1.81; 95% CI, 1.20–2.75), but not significantly in women (OR, 1.56; 95% CI, 0.72–3.41), potentially reflecting lower power or residual confounding. Clinically, these findings support evaluation for severe or familial lipid disorders and intensive LDL-C lowering in patients with ASCVD and LDL-C ≥ 190 mg/dL, particularly with premature disease or family history [4,18,28].

Premature ASCVD also showed lower hypertension prevalence and a weaker association with diabetes than later-onset disease. Adjusted analyses showed inverse associations of hypertension with premature presentation in both sexes and of diabetes in men. Given that age is embedded in the outcome definition, these findings likely reflect greater accumulation of age-related comorbidity in later-onset disease rather than protective effects.

Overall, the findings support a shared risk-accumulation trajectory rather than distinct disease processes: premature ASCVD appears more strongly related to high-intensity early exposures, including smoking and inherited or acquired lipid abnormalities, whereas later-onset disease reflects longer accumulation of hypertension and diabetes. Because conventional short-term risk calculators are strongly age-dependent, they may underestimate risk in younger individuals with substantial smoking, family history, or dyslipidemia [4,5]. Multimodality imaging may help identify subclinical disease and support lifetime anatomical risk stratification [30]. European cohort studies similarly show that younger individuals with adverse risk profiles may remain below 10-year treatment thresholds despite substantial potential benefit from early prevention, supporting the use of lifetime-risk approaches alongside conventional calculators [31].

### 4.5. ACS as the Dominant Presentation and the Role of Plaque Vulnerability

ACS was the predominant ASCVD presentation in both groups and was associated with premature ASCVD in men but not women. Given the coronary-dominant cohort, these findings primarily apply to ACS and may not generalize less-represented phenotypes such as PAD and cerebrovascular disease.

From a pathobiological point of view, younger patients with ASCVD may be more likely to present with acute thrombotic events than with stable obstructive coronary disease. This has been attributed in part to differences in plaque composition, including lipid-rich plaques with thin fibrous caps that are susceptible to rupture [32]. However, the present study did not assess plaque morphology, culprit lesion characteristics, intravascular imaging, or angiographic features of plaque vulnerability. Therefore, the relationship between the higher atherogenic lipid burden observed in premature ASCVD and plaque vulnerability should be considered biologically plausible but hypothesis-generating rather than directly demonstrated by the current data.

### 4.6. Obesity, Chronic Kidney Disease, and Heart Failure in Supplementary Models

Further analysis of the supplemental clinical subset models that applied to only a sub-cohort also yielded further insights. There was a significant positive association between obesity and overweight and early ASCVD onset among men, and there was an inverse relationship with early ASCVD in the presence of chronic kidney disease and heart failure among women. The relationship of obesity with premature ASCVD among men is congruent with the global trend of early onset of the cardiometabolic syndrome among men who have increasing obesity, which adds to their risk factors of dyslipidemias and smoking, thus resulting in faster atherosclerosis [33]. On the other hand, the inverse relationships between chronic kidney disease and heart failure with early ASCVD onset may be attributed to their higher prevalence among older patients with a history of cardiovascular and metabolic diseases.

### 4.7. Secondary Prevention Medication Use and Residual Risk

Recorded secondary prevention medication use was high in both groups, with similar statin (93.4% vs. 94.7%), antiplatelet (96.3% vs. 97.5%), and combined statin–antiplatelet use (91.9% vs. 93.1%) in premature versus later-onset ASCVD. Thus, treatment use did not appear to explain the observed clinical and lipid differences.

Nevertheless, the higher atherogenic lipid burden in premature ASCVD remains clinically important. Median LDL-C was 116.0 mg/dL, and 66.6% had LDL-C ≥ 100 mg/dL. However, 95.4% of measurements preceded lipid-lowering therapy, and information on treatment intensity, adherence, duration, and non-statin therapy was unavailable. Moreover, because most patients were assessed after their index event, these values may not represent premorbid exposure.

These findings indicate a substantial baseline atherogenic burden and underscore the importance of intensive secondary prevention. Current ESC/EAS guidance recommends ≥50% LDL-C reduction and a target <55 mg/dL in very-high-risk patients, typically using high-intensity or maximally tolerated statins, followed by ezetimibe and, when indicated, PCSK9 inhibitors [18]. FOURIER and ODYSSEY OUTCOMES demonstrated additional cardiovascular benefit from PCSK9 inhibition in appropriately selected patients with established ASCVD [34,35]. However, treatment response was not assessed, and the present study cannot determine which patients required treatment intensification.

### 4.8. Clinical and Public Health Implications

Rather than implying that premature ASCVD warrants an entirely separate prevention paradigm, these findings have several implications for earlier and more intensive management of shared, modifiable risk factors in Jordan and similar Middle Eastern settings. First, the strong association between current smoking and premature presentation supports prioritizing tobacco-control policies, systematic assessment of tobacco exposure, and accessible, evidence-based smoking-cessation interventions. Second, the high prevalence of family history and the greater frequency of severe LDL-C elevation among premature cases support careful evaluation for familial or inherited lipid disorders and consideration of cascade lipid screening when familial hypercholesterolemia is clinically suspected. Third, sex-stratified assessment is important because the prevalence and strength of associations differed between men and women, although formal interaction between sex and individual risk factors was not tested.

The findings may also inform future development or evaluation of cardiovascular risk-assessment approaches for Middle Eastern populations.

### 4.9. Strengths and Limitations

This study provides a large assessment of premature ASCVD in 2763 Middle Eastern patients, enabling sex-stratified multivariable analyses. Pooling multiple cardiovascular datasets provided clinical diversity, while harmonized definitions and detailed lipid measurements enabled characterization of clinical and atherogenic profiles. Supplementary models further assessed obesity, chronic kidney disease, and heart failure.

Several limitations should be considered. First, the observational design precludes causal inference, and residual confounding remains possible. Second, age was not adjusted for because it is embedded in the definition of premature ASCVD; consequently, age-related comorbidities may differ partly by design. Third, genetic testing and formal diagnostic criteria for familial hypercholesterolemia were unavailable, precluding estimation of its prevalence.

The cohort predominantly represented coronary disease (97.9%), limiting generalizability to premature cerebrovascular disease and PAD. Although most lipid measurements preceded therapy, 4.6% were obtained 2–4 weeks after statin initiation. Limited information on treatment intensity, duration, adherence, and non-statin therapy may have introduced exposure misclassification and underestimated untreated LDL-C burden. Similarly, smoking and some lipid measurements were obtained at or after the index event, allowing post-event behavior or treatment effects to influence estimates.

BMI, chronic kidney disease, and heart failure were available only in a subset, reducing precision and potentially introducing selection bias. Women-specific factors, including pregnancy-related complications and premature menopause, were unavailable, and female analyses had lower precision for uncommon exposures. Overlap between dyslipidemia and LDL-C ≥ 190 mg/dL may also have introduced shared variance.

Most risk factors were present in both groups, with generally modest between-group differences, suggesting a shared risk-factor architecture rather than distinct etiologies. Female smoking may have been underreported because of social stigma, and smoking status did not distinguish cigarettes from waterpipe use.

Finally, the pooled cohort was heterogeneous and not population-based. Inclusion of a study targeting young Middle Eastern women likely contributed to the 66.4% proportion of premature ASCVD among women; this should not be interpreted as a regional prevalence. The post-revascularization cohort may also have introduced survivorship and selection bias, although age at the original event minimized age-group misclassification.

## 5. Conclusions

In this Middle Eastern cohort, premature and later-onset ASCVD shared a largely overlapping risk-factor profile but differed in the relative burden and predominance of individual risk factors, consistent with a model of cumulative exposure over time rather than two distinct disease entities. Premature presentation was disproportionately associated with current smoking, family history of premature cardiovascular disease, and atherogenic dyslipidemia, exposures capable of acting from early adulthood, while later-onset presentation carried a greater burden of age-accumulated metabolic disease, including hypertension, diabetes, and chronic kidney disease. These findings primarily describe a predominantly coronary cohort, particularly patients with ACS, and should not be generalized to premature PAD or cerebrovascular disease without confirmation in populations with adequate representation of these conditions.

Across both sexes, current smoking was the strongest and most consistent correlate of premature presentation, while family history and elevated LDL-C pointed to an additional contribution of inherited and metabolic risk. These findings support further evaluation of early smoking-cessation efforts and targeted lipid assessment in patients with premature ASCVD, rather than a fundamentally different prevention paradigm—consistent with early- and later-onset disease following a shared risk-accumulation pathway rather than separate disease processes.

## Figures and Tables

**Table 1:** Characteristics of Cardiovascular Registries and Investigations Conducted in Jordan and the Middle East.

Study/Registry	ClinicalTrials.gov Identifier	Primary Objective	Study Duration	Inclusion Parameters	Exclusion Parameters	Study Population Characteristics	Participating Institutions
Jordan SMuRF-less Study	NCT06199869	Characterize CV risk beyond SMuRFs	10 January 2024–20 August 2024	Adults ≥18 with ASCVD	Congenital anomalies, pregnancy/lactation, no consent	Jordanian ASCVD patients with emerging risks	9 healthcare facilities across Jordan
First Jordan PCI Registry	NCT01841346	Evaluate PCI outcomes and complications	Ongoing	Adults ≥18 undergoing PCI for coronary artery disease (CAD)	PCI contraindications, refusal, severe comorbidities	Elective and emergent PCI patients	Multiple PCI-capable hospitals in Jordan
ASCVD Risk Factors in Young Women	NCT04975503	Identify traditional and novel ASCVD risks	Long-term	Women 18–45 with or at risk of ASCVD	Pregnancy, CABG, major CV events, chronic illness	Young Middle Eastern women at CV risk	Urban centers across the Middle East
Survival Post-Coronary Revascularization	NCT03491722	Assess long-term survival post-revascularization	>10-year follow-up	Adults surviving ≥10 years post PCI/CABG	Recent procedures, terminal illness	Long-term CAD survivors	Hospitals across the Middle East
Jordan Atrial Fibrillation Study	NCT03917992	Assess Atrial fibrillation (AF) epidemiology and outcomes	Ongoing	Adults ≥18 with AF	Non-cardiac arrhythmias, pregnancy, contraindications	Incident and prevalent AF cases	Jordanian cardiology centers
Statin Eligibility in AMI Patients	NCT03485742	Evaluate statin prescribing and eligibility	Not specified	Adults ≥ 18 with AMI	Statin intolerance or contraindication	AMI patients in the Middle East	High-volume AMI centers
Jordan COVID-19 Acute CV Events Study	NCT04368637	Assess COVID-19 impact on CV events	Pandemic period	Adults ≥18 with acute CV events	No CV events, confounders	Jordanian acute CV cases during COVID-19	Hospitals across Jordan

**Table 2:** Demographic and Clinical Characteristics by Premature ASCVD Status.

Characteristic	Later-Onset ASCVD	Premature ASCVD	SMD	*p* Value
Female	232/1428 (16.2)	458/1335 (34.3)	0.42	<0.001
Age, years	65.0 [59.0–71.0]	49.0 [44.0–53.0]	−2.35	—
Current smoker	569/1428 (39.8)	764/1335 (57.2)	0.35	<0.001
Family history of premature CVD	534/1428 (37.4)	647/1335 (48.5)	0.22	<0.001
Hypertension	957/1428 (67.0)	620/1335 (46.4)	−0.42	<0.001
Dyslipidemia	1215/1428 (85.1)	1102/1335 (82.5)	−0.07	0.070
Diabetes mellitus	836/1428 (58.5)	649/1335 (48.6)	−0.20	<0.001
ACS presentation	1206/1428 (84.5)	1172/1335 (87.8)	0.10	0.011
Statin use	1353/1428 (94.7)	1247/1335 (93.4)	−0.06	0.135
Any antiplatelet	1392/1428 (97.5)	1286/1335 (96.3)	−0.07	0.080
Statin + any antiplatelet	1330/1428 (93.1)	1227/1335 (91.9)	−0.05	0.220
BMI, kg/m^2^	28.0 [25.0–31.2]	29.0 [25.5–32.2]	0.14	<0.001

Values are *n*/N (%) or median [IQR]. ASCVD = atherosclerotic cardiovascular disease; CVD = cardiovascular disease; ACS = acute coronary syndrome; BMI = body mass index; SMD = standardized mean difference. Comparisons used Pearson’s chi-square test for categorical variables and the Mann–Whitney U test for continuous variables, with available data for each variable. BMI was available for 1069 later-onset and 921 premature cases. Age was not tested because it determined group classification. *p* values were two-sided and exploratory, without adjustment for multiple comparisons.

**Table 3:** Lipid Profile by Premature ASCVD Status.

Characteristic	Later-Onset ASCVD	Premature ASCVD	SMD	*p* Value
LDL-C >= 190 mg/dL	57/1428 (4.0)	101/1335 (7.6)	0.15	<0.001
LDL-C >= 100 mg/dL	735/1428 (51.5)	889/1335 (66.6)	0.31	<0.001
Triglycerides >= 150 mg/dL	680/1428 (47.6)	774/1335 (58.0)	0.21	<0.001
Low HDL-C	919/1428 (64.4)	966/1335 (72.4)	0.17	<0.001
LDL-C, mg/dL	100.0 [77.0–128.0]	116.0 [89.0–148.5]	0.36	<0.001
Total cholesterol, mg/dL	164.0 [138.0–193.0]	184.0 [154.0–219.0]	0.39	<0.001
HDL-C, mg/dL	37.0 [31.0–45.2]	36.0 [31.0–44.0]	−0.04	0.029
Triglycerides, mg/dL	145.0 [101.0–201.0]	166.0 [115.5–239.0]	0.22	<0.001
Non-HDL-C, mg/dL	125.0 [99.0–156.0]	145.0 [115.0–181.0]	0.40	<0.001
TC/HDL-C ratio	4.4 [3.5–5.6]	5.0 [3.9–6.3]	0.37	<0.001

Values are *n*/N (%) or median [IQR]. LDL-C = low-density lipoprotein cholesterol; HDL-C = high-density lipoprotein cholesterol; TC = total cholesterol; SMD = standardized mean difference. Comparisons used Pearson’s chi-square test for categorical variables and the Mann–Whitney U test for continuous variables. All lipid comparisons included 1428 later-onset and 1335 premature cases. *p* values were two-sided.

**Table 4:** Sex-Stratified Clinical Profile by Premature ASCVD Status.

Characteristic	Males Later-Onset	Males Premature	*p* Value	Females Later-Onset	Females Premature	*p* Value
Current smoker	554/1196 (46.3)	655/877 (74.7)	<0.001	15/232 (6.5)	109/458 (23.8)	<0.001
Family history of premature CVD	453/1196 (37.9)	436/877 (49.7)	<0.001	81/232 (34.9)	211/458 (46.1)	0.005
Hypertension	752/1196 (62.9)	330/877 (37.6)	<0.001	205/232 (88.4)	290/458 (63.3)	<0.001
Diabetes mellitus	673/1196 (56.3)	378/877 (43.1)	<0.001	163/232 (70.3)	271/458 (59.2)	0.004
Dyslipidemia	1020/1196 (85.3)	708/877 (80.7)	0.006	195/232 (84.1)	394/458 (86.0)	0.488
LDL-C >= 190 mg/dL	47/1196 (3.9)	69/877 (7.9)	<0.001	10/232 (4.3)	32/458 (7.0)	0.165
ACS presentation	1012/1196 (84.6)	797/877 (90.9)	<0.001	194/232 (83.6)	375/458 (81.9)	0.570
Obesity	251/874 (28.7)	185/545 (33.9)	0.038	98/195 (50.3)	193/376 (51.3)	0.808
Heart failure	179/874 (20.5)	94/546 (17.2)	0.129	65/195 (33.3)	65/377 (17.2)	<0.001

Note. Values are *n*/N (%), using the available denominator for each variable. *p* values compare premature and later-onset ASCVD separately within males and females using Pearson’s chi-square test. *p* values were two-sided. ASCVD = atherosclerotic cardiovascular disease; CVD = cardiovascular disease; LDL-C = low-density lipoprotein cholesterol; ACS = acute coronary syndrome.

**Table 5:** Sex-Stratified Adjusted Odds Ratios for Premature ASCVD.

Model	Predictor	OR	95% CI	*p*
Male	Family history of premature CVD	1.71	1.41–2.07	<0.001
	Current smoking	3.03	2.49–3.70	<0.001
	Hypertension	0.41	0.34–0.50	<0.001
	Dyslipidemia	0.69	0.53–0.89	0.004
	Diabetes mellitus	0.81	0.66–0.98	0.031
	LDL-C >= 190 mg/dL	1.81	1.20–2.75	0.005
	ACS presentation	1.54	1.14–2.07	0.005
Female	Family history of premature CVD	1.66	1.17–2.34	0.004
	Current smoking	4.07	2.27–7.30	<0.001
	Hypertension	0.25	0.15–0.39	<0.001
	Dyslipidemia	1.12	0.70–1.80	0.629
	Diabetes mellitus	0.90	0.62–1.31	0.583
	LDL-C >= 190 mg/dL	1.56	0.72–3.41	0.261
	ACS presentation	0.78	0.50–1.22	0.271

Note. Core models included family history of premature cardiovascular disease, current smoking, hypertension, dyslipidemia, diabetes mellitus, LDL-C >= 190 mg/dL, and ACS presentation. OR = odds ratio; CI = confidence interval.

## Data Availability

The data that support the findings of this study are available from the corresponding author upon reasonable request.

## Supplementary Materials

The following supporting information can be downloaded at: https://www.mdpi.com/article/10.3390/diagnostics16203354/s1, Supplementary Table S1: Recorded diagnostic categories by premature ASCVD status; Supplementary Table S2: Secondary prevention and other medications by premature ASCVD status; Supplementary Table S3: Sex-stratified clinical subset models for premature ASCVD.

## Abbreviations

ACC	American College of Cardiology
ACS	Acute coronary syndrome
AF	Atrial fibrillation
AHA	American Heart Association
AMI	Acute myocardial infarction
ASCVD	Atherosclerotic cardiovascular disease
BMI	Body mass index
CABG	Coronary artery bypass graft
CAD	Coronary artery disease
CCTA	Coronary computed tomography angiography
CI	Confidence interval
CKD	Chronic kidney disease
CV	Cardiovascular
CVA	Cerebrovascular accident
CVD	Cardiovascular disease
EAS	European Atherosclerosis Society
ESC	European Society of Cardiology
FH	Familial hypercholesterolemia
HDL-C	High-density lipoprotein cholesterol
IQR	Interquartile range
LDL-C	Low-density lipoprotein cholesterol
NCEP ATP III:	National Cholesterol Education Program Adult Treatment Panel III
non-HDL-C	Non-high-density lipoprotein cholesterol
OR	Odds ratio
PAD	Peripheral artery disease
PCI	Percutaneous coronary intervention
PCSK9	Proprotein convertase subtilisin/kexin type 9
SMD	Standardized mean difference
SMuRF	Standard modifiable risk factor
TC	Total cholesterol
VIF	Variance inflation factor
