# Supplementary material for: Premature Atherosclerotic Cardiovascular Disease in a Middle Eastern Cohort: A Risk-Accumulation Perspective on Shared Determinants and Temporal Exposure Patterns
Source: Diagnostics (Basel). 2026 Oct 15;16(20):3354. doi: 10.3390/diagnostics16203354 (PMC13702967; doi:10.3390/diagnostics16203354)
Supplement: Supplementary file 1 [file diagnostics-16-03354-s001.zip › diagnostics-4527531-supplementary.pdf]

Supplementary tables

Premature Atherosclerotic Cardiovascular Disease in a Middle Eastern Cohort: A Risk-Accumulation Perspective on Shared Determinants and Temporal Exposure Patterns

Supplementary Table S1. Recorded diagnostic categories by premature ASCVD status

Recorded diagnosis	Later-onset ASCVD (n = 1,428)	Premature ASCVD (n = 1,335)	Overall (n = 2,763)
Acute coronary syndrome	1206 (84.5)	1172 (87.8)	2378 (86.1)
Cerebrovascular accident	20 (1.4)	28 (2.1)	48 (1.7)
CAD diagnosed by CCTA	51 (3.6)	15 (1.1)	66 (2.4)
Peripheral artery disease	4 (0.3)	6 (0.4)	10 (0.4)
Chronic stable angina	147 (10.3)	114 (8.5)	261 (9.4)
Total	1428 (100.0)	1335 (100.0)	2763 (100.0)

Values are n (%), with percentages calculated within each column. The diagnostic field recorded one category per participant; these frequencies do not represent the prevalence of all coexisting vascular conditions. The cerebrovascular accident category was retained as recorded because the analytical dataset did not separately establish ischemic subtype. Carotid atherosclerosis was not separately coded and could not be quantified independently. ASCVD = atherosclerotic cardiovascular disease; CAD = coronary artery disease; CCTA = coronary computed tomography angiography.

Supplementary Table S2. Secondary prevention and other medications by premature ASCVD status

Medication	Later-onset ASCVD	Premature ASCVD	SMD
Aspirin	1339/1428 (93.8)	1263/1335 (94.6)	0.04
Clopidogrel	771/1424 (54.1)	690/1334 (51.7)	-0.05
Ticagrelor/prasugrel	278/1424 (19.5)	260/1333 (19.5)	0.00
Dual antiplatelet therapy	1004/1428 (70.3)	924/1335 (69.2)	-0.02
Statin	1353/1428 (94.7)	1247/1335 (93.4)	-0.06
RAAS inhibitor	836/1428 (58.5)	739/1335 (55.4)	-0.06
Beta-blocker	1047/1428 (73.3)	945/1333 (70.9)	-0.05
Oral hypoglycemic agent	272/1069 (25.4)	196/923 (21.2)	-0.10
Insulin	250/1069 (23.4)	188/923 (20.4)	-0.07

Values are n/N (%) using the available denominator for each medication. SMDs were calculated as premature minus later-onset ASCVD. ASCVD = atherosclerotic cardiovascular disease; SMD = standardized mean difference; RAAS = renin–angiotensin–aldosterone system.

Supplementary Table S3. Sex-stratified clinical subset models for premature ASCVD

Predictor	Males OR (95% CI)	P value	Females OR (95% CI)	P value
Family history of premature CVD	1.81 (1.43–2.29)	<0.001	1.59 (1.05–2.40)	0.029
Current smoking	3.17 (2.49–4.03)	<0.001	3.76 (1.81–7.82)	<0.001
Hypertension	0.48 (0.38–0.62)	<0.001	0.40 (0.22–0.71)	0.002
Dyslipidemia	0.59 (0.44–0.78)	<0.001	0.96 (0.56–1.64)	0.872
Diabetes mellitus	0.78 (0.61–0.99)	0.038	1.53 (0.95–2.46)	0.081
LDL-C ≥190 mg/dL	1.35 (0.78–2.31)	0.281	1.76 (0.61–5.05)	0.294
ACS presentation	1.39 (1.02–1.89)	0.039	0.79 (0.47–1.31)	0.355
Overweight	1.45 (1.07–1.97)	0.018	0.62 (0.32–1.21)	0.161
Obesity	1.86 (1.34–2.59)	<0.001	1.00 (0.53–1.87)	0.993
Chronic kidney disease	0.44 (0.25–0.78)	0.005	0.56 (0.27–1.16)	0.120
Heart failure	0.91 (0.67–1.23)	0.528	0.59 (0.36–0.96)	0.034

Male model: n = 1,419, including 545 premature cases; female model: n = 429, including 234 premature cases. Models used complete cases for all included variables and simultaneously adjusted for every predictor shown. The outcome was premature versus later-onset ASCVD. Overweight and obesity were entered as separate indicator variables, with the reference category comprising participants classified as neither overweight nor obese. OR = adjusted odds ratio; CI = confidence interval; CVD = cardiovascular disease; LDL-C = low-density lipoprotein cholesterol; ACS = acute coronary syndrome; ASCVD = atherosclerotic cardiovascular disease.
